# Daily Heart Rate Variability in Dogs With Atrial Fibrillation

**DOI:** 10.1111/jvim.70051

**Published:** 2025-03-08

**Authors:** Joao Escalda, Brigite Pedro, Jose Novo Matos, Antonia Mavropoulou, Christopher Linney, Joao Neves, Joanna Dukes‐McEwan, Anna R. Gelzer

**Affiliations:** ^1^ The Queen's Veterinary School Hospital University of Cambridge Cambridge UK; ^2^ Willows Veterinary Centre and Referral Service West Midlands UK; ^3^ Hospital Veterinário Do Bom Jesus Braga Portugal; ^4^ Centro de Cardiologia Veterinária Do Atlântico Mafra Portugal; ^5^ Virtual Veterinary Specialists Ltd Middlesex UK; ^6^ Plakentia Veterinary Clinic Athens Greece; ^7^ Paragon Veterinary Referrals Wakefield UK; ^8^ Small Animal Teaching Hospital, Department of Small Animal Clinical Science University of Liverpool Leahurst Campus UK; ^9^ Department of Clinical Studies and Advanced Medicine, School of Veterinary Medicine University of Pennsylvania Philadelphia Pennsylvania USA

**Keywords:** antiarrhythmic therapy, electrocardiography, holter, rate control, ventricular arrhythmias

## Abstract

**Background:**

Daily variability of heart rate in 24‐h Holter recordings in dogs with atrial fibrillation (AF) receiving antiarrhythmic drugs (AAD) is unknown and could influence medical decisions.

**Hypothesis/Objectives:**

Dogs with AF, Holter‐derived mean heart rate (meanHRHolter) over 24 h is not significantly different from a subsequent, consecutive 24‐h period.

**Animals:**

Twenty‐five dogs with AF.

**Methods:**

Prospective, descriptive, multicenter study. MeanHRHolter rate and ventricular arrhythmias (VAs) were prospectively analyzed after starting AAD. Clinically relevant difference (defined as ≥ 10 bpm in the meanHRHolter), success of rate control (defined as meanHRHolter ≤ 125 bpm). A Bland–Altman analysis and intra‐class correlation coefficient (ICC) were calculated to compare two consecutive 24‐h Holter recordings. VAs percentage difference [(maximum daily value‐minimum daily value)/maximum daily value × 100] and grading variability between recordings were also investigated.

**Results:**

Small BIAS with ICC 0.98 (95% confidence interval [CI] 0.95–0.99) on meanHRHolter with no statistical difference between two consecutive 24‐h Holter recordings (95% CI [−2.84–2.92], degree of freedom 24, *p* = 0.98). Only 2/25 dogs (8%; 95% CI [2%–25%]) had clinically significant variation, while 1/25 (4%; 95% CI [0%–20%]) dogs showed different classifications in the success of rate control between the consecutive recordings. The VAs percentage difference was 52%, with 7/25 (28%; 95% CI [14%–47%]) dogs showing a VAs grading difference of ≥ 2.

**Conclusion and Clinical Importance:**

The daily heart rate variability in dogs with AF receiving AAD is low, suggesting that a single 24‐h Holter recording is adequate to assess rate control. Daily variability might be an important consideration when assessing VAs in dogs with concomitant AF.

**Abbreviations**
24‐h Holter_1_
: first 24‐h Holter
24‐h Holter_2_
: second 24‐h Holter
AAD: anti‐arrhythmic drugs
AF: atrial fibrillation
ARVC: arrhythmogenic right ventricular cardiomyopathy
CHF: congestive heart failure
CV: coefficient of variation
HR: heart rate
HR_ECG_
: in‐clinic electrocardiography‐derived HR
ICC: intraclass correlation coefficient
MeanHR_Holter_
: Holter‐derived mean HR
VAs: ventricular arrhythmias

## 1 Introduction

Atrial fibrillation (AF) is the most common non‐physiological arrhythmia in dogs, well known for being a negative prognostic factor [1, 2, 3, 4, 5]. Most dogs affected by AF have advanced cardiac remodeling at the time of diagnosis, making rhythm control an unrewarding strategy [6]. Therefore, rate control is most commonly recommended [5, 7, 8, 9, 10, 11, 12]. The association of heart rate (HR) with survival time has been established [1, 5, 13]. Dogs in AF with an in‐clinic electrocardiography‐derived HR (HR_ECG_) < 160 beats per minute (bpm) show longer survival times than dogs with an HR_ECG_ > 160 bpm [13]. However, these dogs are subjected to a stress‐related high‐sympathetic drive, which might increase the HR [14], without any clear correlation with a Holter‐derived HR [15], obtained in their home environment, which is considered a more reliable method to assess HR in dogs with AF [1]. Achieving appropriate rate control, defined as a Holter‐derived mean HR (meanHR_Holter_) ≤ 125 bpm, improves survival in dogs with AF [1, 5]. Moreover, for each increment of 10 bpm in meanHR_Holter_, there is an increase of 35.5% in the risk of cardiac death over a 12‐month period [1]. Hence, meanHR_Holter_ is currently the recommended method to assess rate control success and dictates clinical decisions on tailoring anti‐arrhythmic therapy.

However, whether a day‐to‐day variability in meanHR_Holter_ exists in dogs with AF has not been investigated. In Boxers with arrhythmogenic right ventricular cardiomyopathy (ARVC) and frequent ventricular arrhythmias (Vas; defined as > 500 VAs/day), day‐to‐day variability in the frequency of VAs was as high as 80%, and nearing 100% in dogs with reduced VAs frequency (defined as < 500 VAs/day) [16]. Also, in Dobermans, spontaneous day‐to‐day variability in the frequency of VAs is close to 100%, even in dogs with frequent VAs [17]. A 4‐day Holter might be more sensitive than a 24‐h Holter at predicting dilated cardiomyopathy based on VAs count criteria [17]. In contrast, comparing the likelihood of arrhythmias in 24‐h versus 48‐h Holter suggested that an additional 24 h of Holter recording increases the likelihood of the identification of a relevant arrhythmia by 5.4% [18], similar to findings in humans [19].

The present study aimed to prospectively investigate the day‐to‐day variability in the ventricular response rate of dogs with AF receiving anti‐arrhythmic drugs (AAD) as part of a rate control strategy. The primary objective was to compare two consecutive 24‐h Holter recordings to determine if the success of rate control (defined as meanHR_Holter_ ≤ 125 bpm) would vary between the first 24 h (24‐h Holter_1_) and the second 24 h (24‐h Holter_2_) as well as investigate overall day‐to‐day variability in meanHR_Holter_. Based on survival studies with similar results [1, 5], we hypothesized that the meanHR_Holter_ of 24‐h Holter_1_ and 24‐h Holter_2_ would not be significantly different. Additionally, we sought to determine the daily variability in the number and grading of VAs in dogs with AF and concurrent cardiac disease.

## 2 Materials and Methods

The Optimal Rate Control in Dogs with Atrial Fibrillation (ORCA) study was a prospective, descriptive, multicenter study. A complete description of the study has been published [1]. Eligible dogs had newly diagnosed AF; other inclusion and exclusion criteria are described [1]. In summary, only dogs with newly diagnosed AF and not receiving any anti‐arrhythmic therapy could be enrolled in the study. Echocardiography, 6‐lead ECG, complete blood work, and measurement of blood pressure were performed on all dogs at initial admission.

At initial admission, if HR_ECG_ was > 150 bpm, AAD for rate control was initiated. This HR cut‐off was selected because HR_ECG_ > 150 bpm in dogs is typically associated with meanHR_Holter_ > 140 bpm [15]. If the HR_ECG_ was ≤ 150 bpm, a Holter monitor was placed before AAD therapy to assess meanHR_Holter_ and determine the requirement for rate control (meanHR_Holter_ > 125 bpm) [1, 5]. Dogs returned to the clinic 1–3 weeks later for Holter monitor placement to assess the success of rate control.

Only dogs with good quality 48‐h Holter recordings that were started on AAD as part of a rate control strategy were included in the present study. In order to reduce confounding potential acclimatization factors, sequential Holter recordings acquired to re‐assess rate control after AAD adjustments were not included in this study (only the first Holter after starting AAD was analyzed). Dogs with congestive heart failure (CHF) were only included if CHF was considered controlled at the time of Holter recording, based on the absence of clinical signs (increased resting respiratory rate, respiratory effort or abdominal distension), echocardiographic (presence of B‐lines with concomitant left atrial enlargement), radiographic changes consistent with right or left‐sided CHF (ascites and perihilar interstitial/alveolar pattern, respectively) or a combination of these findings. Dogs were excluded if changes in CHF therapy were required before the Holter placement, in order to minimize variations in sympathetic tone.

Holters were acquired using digital 3‐lead monitors (Lifecard CF, Spacelabs Healthcare Ltd) and the analyses were performed by a single board‐certified cardiologist using a commercially available analysis software (Pathfinder 9.0, Spacelabs Healthcare Ltd). The meanHR_Holter_, minimum and maximum HR, as well as the number and complexity of VAs, were tabulated and analyzed for each 24 h period (24‐h Holter_1_ and 24‐h Holter_2_). Heart rate variables were calculated based on a minute average. Dogs were classified as “rate‐controlled” if the meanHR_Holter_ was ≤ 125 bpm. Based on the increased risk of cardiac death for every 10 bpm in the meanHR_Holter_ [1], a difference ≥ 10 bpm in the meanHR_Holter_ between 24‐h Holter_1_ and 24‐h Holter_2_ was considered clinically relevant. If VAs were recorded, they were graded based on complexity [16]: Grade 0 = no VAs; Grade 1 = single VAs only; Grade 2 = VAs as bigeminy, trigeminy; Grade 3 = presence of VAs and couplets or triplets; and Grade 4 = R‐on‐T phenomenon or ventricular tachycardia (≥ 4 consecutive VAs) [16]. The Holter recordings were reviewed by a cardiologist to differentiate Ashman phenomenon from VAs [20].

For this study, a variability of ≥ 2 grades was considered clinically relevant as it could influence the selection of AAD. Dogs with a high number of VAs (> 500/24 h) and a low number of VAs (< 500/24 h) were analyzed separately, as reported [16].

## 3 Statistical Methods

Statistical analyses were performed using commercially available software IBM SPSS Statistics version 28.0. and R Core Team (2024) *“*R: A Language and Environment for Statistical Computing. R Foundation for Statistical Computing, Vienna, Austria”. Continuous data were assessed for normality using the Shapiro–Wilk Test and QQ plots. Normally distributed data are summarized as means and (standard deviations). Non‐normally distributed data are summarized as medians and [25th and 75th quantiles]. Bland–Altman analyses were used to assess the difference between minimum, mean and maximum heart rate between 24‐h Holter_1_ and 24‐h Holter_2_. This is reported as BIAS (mean difference), limits of agreement and confidence intervals (CI). Intraclass correlation coefficient (two‐way random effects, absolute agreement, single rater/measurement; ICC 2,1) was also calculated for minimum, mean and maximum heart rate as well as total VAs between 24‐h Holter_1_ and 24‐h Holter_2_.

A paired sample T‐test or Wilcoxon signed‐rank test, as appropriate, was performed comparing the meanHR_Holter_, maximal heart rate, and minimal heart rate between 24‐h Holter_1_ and 24‐h Holter_2_.

The percentage difference [(maximum daily value‐minimum daily value)/maximum daily value × 100] [16] was calculated for the VAs between both 24 h‐Holter periods. The VAs analysis was repeated separately for dogs with frequent VAs (≥ 500 VAs/24 h) and for dogs with infrequent VAs (< 500 VAs/24 h) [16].

Descriptive analysis was conducted for each 24‐h Holter period to examine the number of dogs with rate control (meanHR_Holter_ < 125 bpm), as well as those with clinically significant differences in the meanHR_Holter_ (defined in this study as a difference ≥ 10 bpm in the mean HR Holter) and significant variations in VPCs grade (defined in this study as a difference ≥ 2 grades). Proportions are reported with a 95% CI.

## 4 Results

A total of 30 dogs met the criteria to be included in this study. Five dogs were excluded because CHF was not considered controlled at the time of the Holter placement, and no dog was excluded due to poor Holter recording quality.

Twenty‐five dogs were included in the present study, with a median age of 9 years [6.4; 12.7] and a mean body weight of 35 kg (± 20.0) comprised the study group. Males were over‐represented (21/25; 84%). Crossbreed (*n* = 6) and Dogue de Bordeaux (*n* = 4) were the most common breeds, followed by Cavalier King Charles Spaniel, Doberman, German Shepherd, Great Dane (*n* = 2 for each breed) and Cocker Spaniel, English Bulldog, Old English Sheepdog, Irish Setter, Labrador, Newfoundland, and Poodle (*n* = 1 for each breed). Thirteen of the 25 (52%) dogs were diagnosed with myxomatous mitral valve disease, 9/25 (36%) with dilated cardiomyopathy, and 3/25 (12%) with congenital heart disease (2 dogs with mitral dysplasia, 1 dog with tricuspid dysplasia). At presentation, 22/25 (88%) dogs were in CHF (8 dogs were in left‐sided CHF, 1 in right‐sided CHF, 12 had bilateral CHF) and 3/25 (12%) were considered to have pre‐clinical disease.

Before starting AAD, a Holter monitor was performed in one dog (HR_ECG_ ≤ 150 bpm at presentation), showing a mean HR Holter of 133 bpm. Dual therapy with diltiazem and digoxin was the most common treatment (17/25 [68%] dogs), followed by diltiazem alone (8/25 [32%] dogs). The Holter monitor was recorded a median of 11 days (8–16) after the introduction of AAD.

**FIGURE 1:**
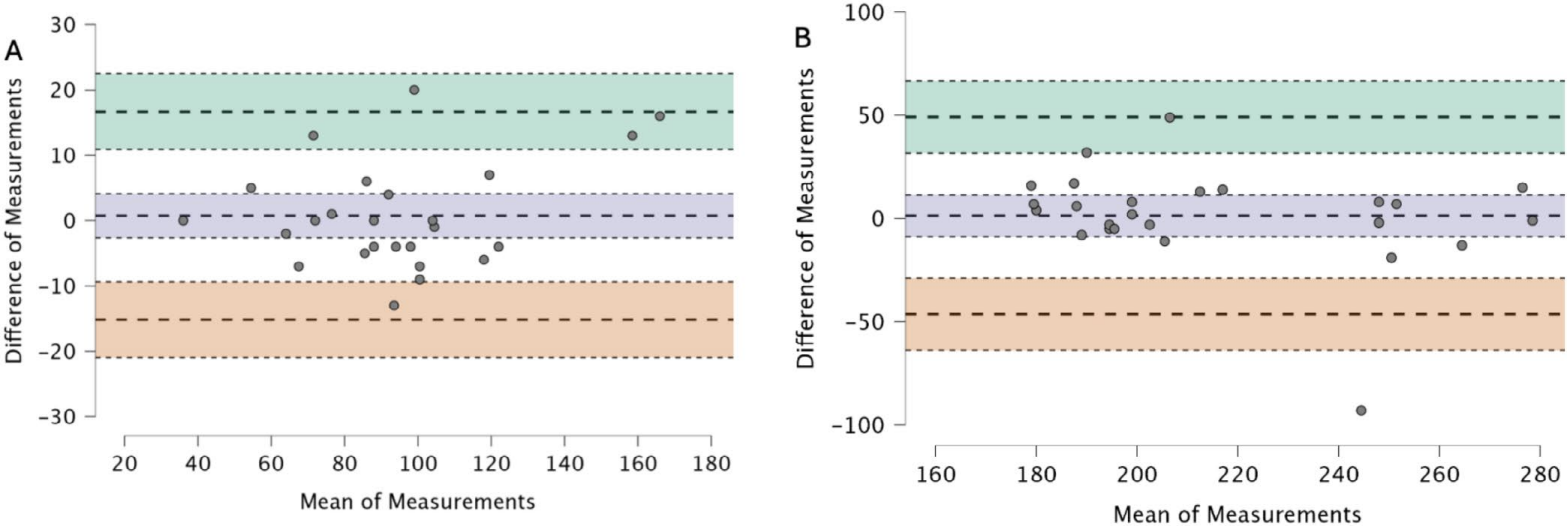
Bland–Altman plots showing minimal (A) and maximal heart rate (B) between the first 24‐h and the second 24‐h Holter. The upper limit of agreement, with a 95% confidence interval, is presented in green; BIAS, with a 95% confidence interval, is presented in blue; and the lower limit of agreement, with a 95% confidence interval, is presented in red. The *Y*‐axis shows the difference in the first 24‐h and second 24‐h Holter (Difference of Measurements), and the *X*‐axis shows the mean measurement for the first 24‐h and second 24‐h (mean of Measurements).

**FIGURE 2:**
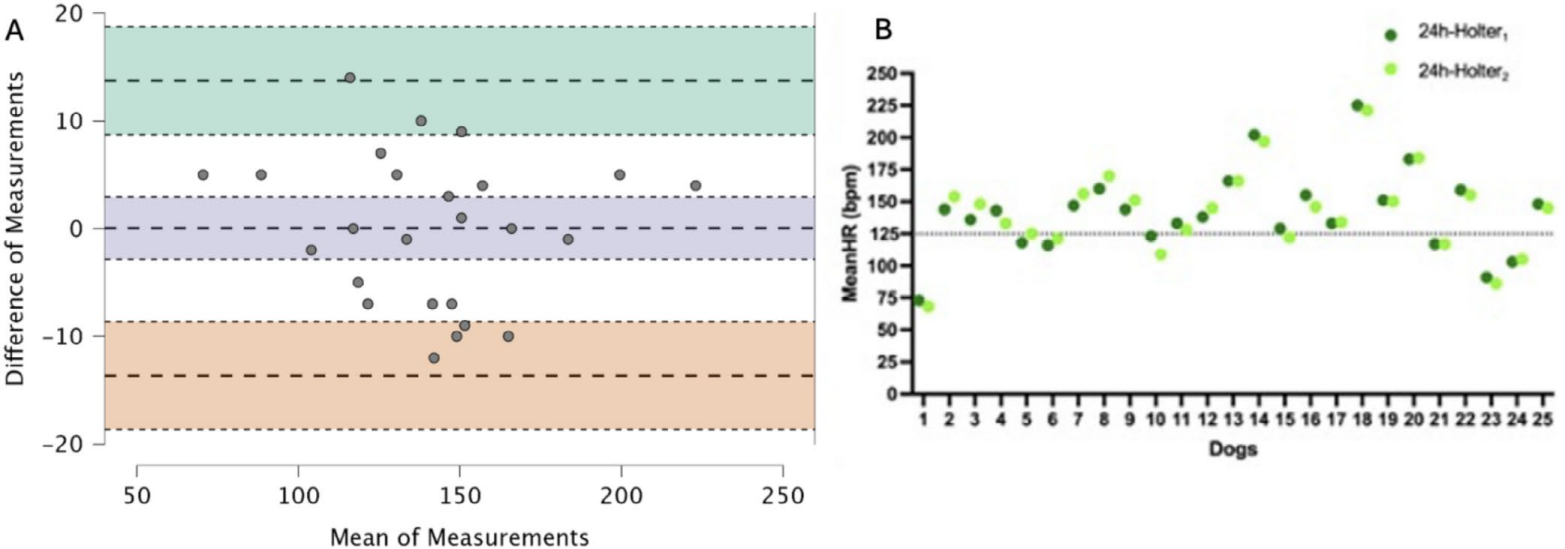
Bland–Altman plots showing mean heart rate (A) and scatterplot (B) for the mean heart rate obtained from a 24‐h Holter (meanHR_Holter_). (A) The upper limit of agreement, with a 95% confidence interval, is presented in green; BIAS, with a 95% confidence interval, is presented in blue; and the lower limit of agreement, with a 95% confidence interval, is presented in red. The *Y*‐axis shows the difference in the first 24‐h and second 24‐h holter (Difference of Measurements), and the *X*‐axis shows the mean measurement for the first 24‐h and second 24‐h (mean of measurements). (B) Scatterplot shows each of the 25 dogs included in the study, during the first 24‐h (24‐h Holter_1_, dark green) and second 24‐h (24‐h Holter_2_, light green).

The results of the Bland Altman analyses are provided in Table 1. The same analyses are presented graphically in Figures 1 and 2. The intraclass correlation coefficient (2,1) for the mean HR Holter was 0.98 (95% CI [0.95–0.99]), for the minimum HR was 0.96 (95% CI [0.92–0.98]), for the maximum HR was 0.76 (95% CI [0.52–0.88]) and for the total VAs was 0.99 (95% CI [0.99–0.1]).

**TABLE 1:** Results of Bland–Altman analysis of the minimal, mean, maximal heart rates (HR) and ventricular arrhythmias (VAs) number between the first 24‐h and the second 24‐h Holter, shown as bias, limits of agreement (LoA) and associated 95% confidence intervals (95% CI).

	BIAS (95% CI)	Lower LoA (95% CI)	Upper LoA (95% CI)
Min HR	1 (−3 to 4)	−15 (−21 to −9)	17 (11−23)
Mean HR	1 (−3 to 3)	−13.65 (−18.64 to −8.66)	14 (9−19)
Max HR	1 (−9 to 11)	−46.33 (−63.73 to −28.92)	49 (31−66)
VAs number	119 (−355 to 593)	−2131 (−2952 to −1310)	2370 (1549−3191)

Abbreviation: LoA = limit of agreement.

There was no significant difference between the minimum HR (95% CI [−2.59–4.1], degree of freedom 24, *p* = 0.64), the maximum HR (*w* = 206, 95% CI [−0.17–0.62], *p* = 0.25) and the mean HR Holter (95% CI [−2.84–2.92], degree of freedom 24, *p* = 0.98) between 24‐h Holter_1_ and 24‐h Holter_2_ (Table 2).

**TABLE 2:** Comparison of minimal, mean, and maximal heart rate (HR) in beats per minute between the first 24‐h and the second 24‐h Holter recordings (24‐h Holter_1_ and 24‐h Holter_2_, respectively). Data are presented as mean (standard deviation) with normal distribution or median [interquartile range] for non‐normal data distribution, with Shapiro–Wilk normality results presented below with degree of freedom (df).

Holter (*N* = 25)	24‐h Holter_1_	24‐h Holter_2_	*p*
Minimal HR	95.4 (± 30.71) df 24 *p* = 0.08	93.9 (± 28.34) df 24 *p* = 0.64	*p* = 0.64
Mean HR	141.5 (± 32.39) df 24*p* = 0.53	141.4(±33.09) df 24 *p* = 0.89	*p* = 0.98
Maximal HR	201 [192–244] df 24 *p* = 0.01	198 [183–248.50] df 24 *p* = 0.01	*p* = 0.25

In 2/25 (8%; 95% CI [2%–25%]) dogs, a variability greater than 10 bpm in meanHR_Holter_ was found between 24‐h Holter_1_ and 24‐h Holter_2_. However, this difference would not have affected the management of these two cases, as both recordings were classified as either rate‐controlled in one case (meanHR_Holter_ 24‐h Holter_1_ 123 bpm; meanHR_Holter_ 24‐h Holter_2_ 109 bpm) or non‐rate‐controlled in the second case (meanHR_Holter_ 24‐h Holter_1_ 136 bpm; meanHR_Holter_ 24‐h Holter_2_ 148 bpm).

Six (24%) dogs were classified as rate‐controlled during 24‐h Holter_1_. In one dog (1/25 [4%; 95% CI [0%–20%]]), rate control classification differed between the two recordings (meanHR_Holter_ 24‐h Holter_1_ 129 bpm, meanHR_Holter_ 24‐h Holter_2_ 122 bpm), an adjustment of the AAD regimen might have been recommended (dog number 15 in Figure 2), the difference in meanHR_Holter_ between 24‐h Holter_1_ and 24‐h Holter_2_ was not considered clinically relevant (< 10 bpm).

The percentage of day‐to‐day variability in the number of VAs of the entire study sample was 52%, with 7/25 (28%; 95% CI [14%–47%]) dogs showing a clinically relevant variability in the VAs grade.

Seventeen (17/25, 68%) dogs had < 500 VAs during the 24 h‐Holter. The percentage of variability in the number of VAs was 64%. Seven out of 17 (41.17%; 95% CI [21%–64%]) dogs showed a clinically relevant difference in the VAs grade. Of these, five dogs (5/7) showed a higher grade during the 24‐h Holter_2_, including one dog changing from a Grade 0 to a Grade 4, and four dogs changing from a Grade 1 to a Grade 3. The other two (2/7) had a higher VAs grade during the 24‐h Holter_1_: one varied from a Grade 4 to a Grade 2, and another from a Grade 3 to a Grade 1.

In the remaining 8/25 (32%) dogs that exhibited ≥ 500 VAs, the percentage difference in the number of VAs was 24%, and none of these dogs displayed a clinically relevant variability in the VAs grade between 24‐h Holter_1_ and 24‐h Holter_2_.

## 5 Discussion

This prospective study investigates HR day‐to‐day variability in dogs with AF receiving AAD as part of a rate control strategy. Our results showed minor BIAS (mean differences) between meanHR_Holter_ between 24‐h Holter_1_ and 24‐h Holter_2_ with no statistically significant differences. This is the most important finding of the present study, as most of the clinical decisions regarding the success of rate control and consequent adjustment of the AAD therapy are currently based on this variable [1, 5]. Considering the low day‐to‐day variability, acquiring multiple consecutive Holter recordings of 24‐h duration does not appear to be superior to a single 24‐h Holter recording to assess the success of rate control therapy in dogs with AF. This supports previous studies, recommending the use of meanHR_Holter_ obtained from a single 24‐h Holter recording to assess the ventricular response rate in dogs with AF, or in other words, the success of rate control [1, 5].

This finding is particularly useful not only in the clinical management of these cases but also from a practical point of view, as shorter recordings might be less uncomfortable for the dogs (which would have to wear the Holter for shorter periods of time), less time‐consuming to analyze, and less expensive than longer recordings.

Nevertheless, 2/25 (8%; 95% CI [2%–25%]) dogs revealed a clinically relevant variability in their meanHR_Holter_ between days (defined as a variability of ≥ 10 bpm). This, possibly by chance, did not change their “rate control” classification, but it could potentially influence the management of the case. Knowing that the risk of death increases with meanHR_Holter_ [1], some clinicians might aim for a stricter rate control, particularly in cases where ventricular response rates close to 125 bpm were easily achieved and therefore another adjustment of the AAD might be well tolerated by the dog and easily accepted by the owners. Nevertheless, a clinically relevant variability in meanHR_Holter_ only occurred in 8% of the entire study sample, with the vast majority having a meanHR_Holter_ difference within 5%. Therefore, a single 24‐h Holter recording remains a valuable and reliable option in the vast majority of cases.

We did not find an obvious tendency in the variability of mean HR between days, contrary to a previous study, where a lower HR was observed during 24‐h Holter_2_ [18]. The lower meanHR during the second 24‐h Holter in that study was suggested to be associated with an acclimatization effect. A possible explanation for the disparate outcomes could stem from the inclusion of just one dog with AF in that particular study [18]. It is worth noting that dogs with AF typically exhibit reduced circadian variability compared to those in sinus rhythm [21], suggesting that dogs with AF might experience less influence from the parasympathetic tone and, therefore, possibly acclimatization.

On the other hand, there was a wide day‐to‐day variability in the number of VAs/24 h (52%). Furthermore, the VAs grading changed by two or more grades (defined in our study as clinically relevant) in 7/25 (28%; 95% CI [14%–47%]) of dogs. This did not appear to reflect acclimatization factors, since more dogs (5/7) had a higher grade of VAs during the second 24‐h period.

When the study group was stratified based on the frequency of ventricular arrhythmias (< 500 or ≥ 500 VAs/24 h), as previously described [16], our results show that dogs with a low VAs burden had increased daily variability of the number of VAs compared to dogs with a high VAs burden (64% vs. 24%, respectively). These results are similar to what was previously reported in Boxers with ARVC [16] and are likely due to different proportional increases. In that study, using the same cutoffs, Boxers with ARVC and with high VAs burden had a daily variability in VAs of 80%, while dogs with lower VAs burden had a daily variability in VAs of 100% [16]. In another study in Dobermans with sinus rhythm, the daily variability in VAs was reported to be close to 100% [17]. Our study demonstrates a lower daily variability in VAs in dogs with AF compared to dogs with sinus rhythm. The reason for the lower day‐to‐day variability in VAs identified in dogs with AF is unknown, but the study sample of dogs in this study represents a broad mix of breeds and underlying diseases, compared to studies in Boxers and Dobermans [16, 17]. Furthermore, it is possible that the irregularity associated with AF could be a contributing factor, as well as the reduction in circadian variability and modulation of autonomic tone, as previously described in dogs with AF when compared to sinus rhythm [21]. Studies with longer Holter recordings could be helpful to confirm and further elucidate these findings.

The 7/25 (28%; 95% CI [14%–47%]) dogs that showed clinically relevant variability in the VAs grading had < 500 VAs/24 h (7/17; 41%, 95% CI [21%–64%]). In dogs with a low VAs burden, anti‐arrhythmic therapy specifically targeting ventricular arrhythmia is typically not instituted. Nevertheless, one dog had no VAs recorded during 24‐h Holter_1_, but a fast run of paroxysmal ventricular tachycardia was recorded during 24‐h Holter_2_ (instantaneous rate of up to 327 bpm for 32 beats). The number of dogs with VAs in each group was small, and these findings should be interpreted with caution. Further investigations are warranted to determine if longer recordings are beneficial when evaluating VAs in dogs with concurrent AF.

There are some limitations in this study. The relatively small sample size is a major limitation, especially for subgroup analysis. Additionally, certain possible confounding factors, such as the severity of the underlying cardiac disease, presence of CHF, or medications prescribed, were not controlled for. The dogs' activity levels or temperament were not standardized, which could have influenced the meanHR_Holter_. While all VAs were verified by a cardiology diplomate, aberrant ventricular conduction (Ashman's phenomenon) cannot be entirely excluded for every complex. Furthermore, the AAD regimen and the time frame between the onset of AF and the acquisition of the first Holter were not standardized; however, for the purpose of optimal comparison between 24‐h Holter_1_ and 24‐h Holter_2_, each dog served as its own control. Finally, only a single 48‐h period was recorded, and longer/multiple recordings might have shown different results.

## 6 Conclusions

This prospective study suggests a low daily variability in the meanHR_Holter_ of dogs with naturally occurring AF receiving AAD, supporting the use of a 24‐h Holter to assess rate control in these dogs. Daily variability in the number and grading of VAs needs to be considered separately when deciding on the optimal duration of Holter monitoring in dogs with concomitant AF.

## Disclosure

Authors declare no off‐label use of antimicrobials.

## Ethics Statement

Approved by the Institutional Animal Care and Use Committee at the University of Pennsylvania (806353) and the Animal Health Trust Clinical Research Ethics Committee (22‐2017 E). Authors declare human ethics approval was not needed.

## Conflicts of Interest

Jose Novo Matos serves as Associate Editor for the Journal of Veterinary Internal Medicine. He was not involved in the review of this manuscript. The other authors declare no conflicts of interest.
